# Linking genetic markers and crop model parameters using neural networks to enhance genomic prediction of integrative traits

**DOI:** 10.3389/fpls.2024.1393965

**Published:** 2024-07-30

**Authors:** Florian Larue, Lauriane Rouan, David Pot, Jean-François Rami, Delphine Luquet, Grégory Beurier

**Affiliations:** ^1^ Centre de Coopération Internationale en Recherche Agronomique pour le Développement (CIRAD), Unité Mixte de Recherche, Institut Amélioration Génétique et Adaptation des Plantes méditerranéennes et Tropicales (UMR AGAP), Montpellier, France; ^2^ Unité Mixte de Recherche, Institut Amélioration Génétique et Adaptation des Plantes méditerranéennes et Tropicales (UMR AGAP), Université Montpellier, Centre de Coopération Internationale en Recherche Agronomique pour le Développement (CIRAD), Institut National de Recherche pour l'Agriculture, l'Alimentation et l'Environnement (INRA), Institut Agro, Montpellier, France

**Keywords:** convolutional neural networks, crop growth model, genomic prediction, sorghum, CGM-WGP

## Abstract

**Introduction:**

Predicting the performance (yield or other integrative traits) of cultivated plants is complex because it involves not only estimating the genetic value of the candidates to selection, the interactions between the genotype and the environment (GxE) but also the epistatic interactions between genomic regions for a given trait, and the interactions between the traits contributing to the integrative trait. Classical Genomic Prediction (GP) models mostly account for additive effects and are not suitable to estimate non-additive effects such as epistasis. Therefore, the use of machine learning and deep learning methods has been previously proposed to model those non-linear effects.

**Methods:**

In this study, we propose a type of Artificial Neural Network (ANN) called Convolutional Neural Network (CNN) and compare it to two classical GP regression methods for their ability to predict an integrative trait of sorghum: aboveground fresh weight accumulation. We also suggest that the use of a crop growth model (CGM) can enhance predictions of integrative traits by decomposing them into more heritable intermediate traits.

**Results:**

The results show that CNN outperformed both LASSO and Bayes C methods in accuracy, suggesting that CNN are better suited to predict integrative traits. Furthermore, the predictive ability of the combined CGM-GP approach surpassed that of GP without the CGM integration, irrespective of the regression method used.

**Discussion:**

These results are consistent with recent works aiming to develop Genome-to-Phenotype models and advocate for the use of non-linear prediction methods, and the use of combined CGM-GP to enhance the prediction of crop performances.

## Introduction

The need to develop plant varieties adapted to evolving production scenarios, especially in the face of climate change, necessitates crops to fulfill increasingly complex and diverse requirements, posing a great challenge for breeders. In this context, the pursuit of traits combinations that confer desired crop properties and adaptation is more critical than ever, giving rise to the necessity to enhance multi-criteria or multi-trait breeding (Moeinizade et al., 2020).

Leveraging the complete set of nucleotide diversity distributed across the genome, for predicting breeding values of quantitative traits (Genomic Prediction, GP, Meuwissen et al., 2001) has already demonstrated its effectiveness in breeding programs. This approach has proven instrumental to increase rates of genetic gains and mitigating costs (Hickey et al., 2017). Nevertheless, the growing need to breed for multiple environments (ME), e.g. in response to climate change and better-defined target populations of environments (Chapman et al., 2000), necessitates the adaptation of genomic prediction methodologies to account for the emergence of interactions between genotypes and environments (GxE) (Rincent et al., 2017).

Previous studies tried to tackle GxE within Genomic Selection (GS). For instance, Burgueño et al. (2012) developed multi-environments statistical models. However, these models only consider linear and non-causal environmental effects reducing possible gains in prediction accuracy, especially for complex integrative traits or for environments that significantly differ from those in the calibration set (Rogers and Holland, 2022). Heslot et al. (2014) on the other hand used a Crop Growth Model (CGM) to derive environmental covariates. The incorporation of environmental covariates within the GS framework increased prediction accuracies and decreased prediction variability in unobserved environments compared to standard GS models. Integrating crop models to address GxE, as shown in studies such as those by Heslot et al. (2014), emphasizes the utility of this approach in the described breeding context. Nonetheless, considering a large number of covariates significantly increases the complexity of the problem, rendering it exceedingly challenging to model (Larkin et al., 2019).

Furthermore, given that the target production traits commonly predicted through GP models are typically polygenic (Song and Zhang, 2009) and thus the presence of potential GxE interactions, the decomposition of these traits into elementary traits, as facilitated by CGM (Bustos-Korts et al., 2019), can offer a valuable advantage. The exploration of multi-trait (MT) genomic prediction strategies has been proposed to enhance the predictive accuracy of integrative traits (e.g., Arojju et al., 2020; Gaire et al., 2022; Shahi et al., 2022). Studies have demonstrated that incorporating correlated physiological traits in the training (and/or validation) sets can improve predictive abilities compared to single-trait (ST) genomic prediction approaches. Crop growth models inherently simulate integrative traits based on other secondary traits, their integration into the genomic prediction framework could thus provide an additional dimension to consider. CGM simulate plant non-linear (causal) responses to the environment through model parameters (representing genotypic sensibility to these responses, GxE). They also have the advantage of being able to simulate multiple traits dynamically. Calibrated CGM for a genotype can thus be helpful to predict its performance in unknown environments (e.g.: Larue et al., 2019), but they cannot predict unknown genotypes.

Combining GP with CGM (integrated CGM-GP prediction) could meet the shortcomings of standard GP models and CGM by enabling the prediction of multiple traits, in multiple environments, for unknown genotypes and unknown environments (Technow et al., 2015; Cooper et al., 2016; Onogi, 2022). However, these studies focus on a small number of parameters, physiological processes, and markers, which contrasts with the need to consider increasingly complex multi-traits phenotypes, and the availability of high-throughput genotyping resulting in a large number of markers.

Another drawback of classical GP models is their linear nature. Integrative traits are generally under the impact of multiple genes, rather than relying solely on small numbers of genetic variations. This is commonly referred to as the “missing heritability problem”. If the effects are additive, then “classical” GP models should be able to capture them. However, if there are non-linear interactions between markers (such as epistasis, Zuk et al., 2012), linear models will fail to predict these integrative traits. Addressing these challenges, non-linear regression methods, such as Deep Learning (Pérez-Enciso and Zingaretti, 2019), coupled with the utilization of Graphical Unit Processing (GPU) computing (Carré et al., 2022), present a promising avenue. This contrasts with conventional linear regression methods (e.g., Montesinos-López et al., 2018), offering in addition the potential to substantially reduce the computational time required for evaluating epistasis.

In CGM, however, integrative traits are simulated by interlinking secondary traits under the influence of multiple genotypic input parameters through non-linear equations. When coupled with genetic information through GP, this approach holds the potential to address the missing heritability problem by simulating integrative traits through non-linear equations that consider the combined effects of all genes.

In this paper, we propose a comparison between three genomic prediction approaches to study the extent to which non-linear regression methods and crop growth models can contribute to enhance the prediction of integrative traits. Using linear (LASSO, Bayes C), or non-linear (Convolutional Neural Networks) regression models, a plant complex integrative phenotypic trait (aboveground fresh weight accumulation) is predicted either directly or with an integrated CGM-GP approach.

## Materials and methods

### Phenotypic data

The phenotypic data used in this study are composed of 136 sorghum accessions selected within the Generation Challenge reference set (Billot et al., 2013). Phenotyping was conducted on the PhenoArch high-throughput phenotyping platform in Montpellier, France (Cabrera Bosquet et al., 2015) where all genotypes were followed for 45 days from September to October 2017. The Phenoarch platform is based on a PhenoWare™ system (Lyon, France) composed of a conveyor belt structure of 28 lanes carrying 60 carts of one pot. Plants were grown in polyvinyl chloride (PVC) 9L pots (0.19m diameter and 0.4m high) filled with a 30:70 mixture of a clay and organic compost. Line spacing was of 0.4m and row spacing was of 0.2m. Five grains per pot were sown on August 30^th^, which were subsequently thinned to a single plant two weeks after sowing. Two water treatment scenarios were tested during the late vegetative phase (starting from eight fully expanded leaves): well-watered (WW) plants were kept at 132% of soil humidity (Fraction of Transpirable Soil Water, FTSW of 0.6) and water-deficit (WD) plants were dried-down until 60% of soil humidity (± 5%, FTSW of 0.22).

Within each treatment, genotypes were replicated four times and local interactions were reduced by separating the genotypes into seven classes depending on plant height. To control the environmental heterogeneity of the greenhouse, the 28 rows were separated into four complete blocks defined according to the environmental gradient of the greenhouse (light and temperature, Cabrera-Bosquet et al., 2016). Each block was further divided into seven sub-blocks wherein plant height classes were randomly assigned following a Youden square (28 genotypes per sub-block). A d-optimal design generation software (SAS procedure OPTEX) was used to assign the genotypes to the sub blocks following an alpha-lattice.

All along the experiment, day temperature was kept at 30°C and night temperatures at 23°C. Environmental variables, air temperature (°C), radiation (PFFD, µmol m-2 s-1) and air relative humidity (%) were continuously monitored at eight positions in the greenhouse, above the canopy and recorded every fifteen minutes.

Thirteen pictures (twelve sides and one top) were taken daily of each plant. Seven genotypes (one for each plant height class, at three different growth stages) were used in a side experiment to calibrate a model used to estimate the following traits on all plants: aboveground fresh weight (Biomaerofw), plant leaf area (PLA), and plant height (PHT). Weekly measurements by hand of additional traits were conducted to assess: the number of appeared (App) and ligulated (Lig) leaves on the main stem and the number of tillers (Tillernb). After 45 days, plants were harvested and final measurements were conducted: shoot (Biomaerofw), mainstem (Mainstemfw) and mainstem blade (Bladefw) biomass fresh weight, base mainstem diameter (Stemdiam), and last ligulated leaf length and width (used to compute area of last ligulated leaf, Arealfel). **Table 1** summarizes all measured traits.

**Table 1:** Phenotypic traits measured on the Phenoarch high-throughput phenotyping platform (see https://cropontology.org).

Phenotypic trait	Crop Ontology id	Unit	Frequency	Method	Abbreviation
Number of appeared leaves*	CO_324:0001016	#	Weekly	By hand	APP
Number of ligulated leaves*	CO_324:0001020	#	Weekly	By hand	LIG
Number of tillers*	CO_324:0000344	#	Weekly	By hand	Tillernb
Plant biomass (fresh weight)*	CO_324:0000558	g	Daily	Image + by hand	Biomaerofw
Plant leaf area		mm²	Daily	Image	PLA
Plant height (ligule of the last ligulated leaf)*	CO_324:0000978	mm	Daily	Image	PHT
Mainstem biomass (fresh weight)*	CO_324:0000777	g	At harvest	By hand	Mainstemfw
Tiller biomass (fresh weight)	CO_324:0000784	g	At harvest	By hand	Tillerfw
Area of last ligulated leaf*		mm²	At harvest	By hand	Arealfel
Blade biomass (fresh weight)	CO_324:0000795	g	At harvest	By hand	Bladefw
Stem diameter	CO_324:0000912	mm	At harvest	By hand	Stemdiam

* used for parameter estimation of the Crop Growth Model.

The integrative trait considered for genomic prediction in this study is a Best Linear Unbiased Predictor (BLUP) of above-ground biomass, estimated by a mixed model (Equation 1, modeled using the R package Asreml v3, Butler et al., 2009) and considering all 8 plant replicates (4 replicates per water treatment, two water treatments).


(1) $$\begin{array}{c} y_{ijk}=a_{i}+bn+C_{j}+d_{1}{\text{𝟙}}_{j=1}+d_{2}{\text{𝟙}}_{j=2}+G_{v}+w_{t}+{\left(Gw\right)}_{vt}+H_{ik}\\ +E_{ivt} \end{array}$$

With $a_{i}$ the fixed effect of the replicate, b the fixed competition effect of any neighbor, *n* the number of neighbors, $C_{j}$ the random effect of any row *j* (except the first and second row), $d_{1}$ and $d_{2}$ the fixed effect of the first and second row. ${𝟙}_{j=1}$ and ${𝟙}_{j=2}$ the indicators of the first and second rows: their value is 1 if the pot belongs to the first (respectively second) row, and 0 otherwise. *G_v_* the random genotypic effect of variety *v, w_t_* the fixed effect of the watering treatment t and (*Gw*)*_vt_* their interaction. H_ik_ the between sub-blocks error, i.e. random effect of the sub-block of the *i* ^th^ replicate to which the *k* ^th^ height class was assigned. *E_ivt_* the residual error for variety *v* in replicate *i* of treatment *t*.

This mixed model was the result of a more complete analysis of the data and assumes that it partially compensates for the heterogeneity of the greenhouse by modelling it as row and column effects. The mixed model was selected after exploring several types of variance decomposition. For the estimation of genotypic parameters of the crop model, raw data from the four WW replicate plants were used alongside local weather data (see Cabrera-Bosquet et al., 2016), as environmental effects are formalized inside the crop model (see *Ecomeristem Model*).

### Genotypic data

Genotypic information of the 136 accessions was obtained through Genotyping by Sequencing (GBS). Sequencing libraries were prepared according to the GBS protocol as per Elshire et al. (2011), with the ApeK1 enzyme. Single-end sequencing was performed on an Illumina HiSeq2000 (at the Genotool platform in Toulouse, France). SNP calling was performed using the GATK pipeline. After the SNP calling step, imputation was then realized using Beagle v.4 (Browning and Browning, 2013) with a 1000-SNP window and an overlap of 500 SNP after filtering on missing data per SNP (60% maximum of missing data and 5% maximum for minor allele frequency to keep a SNP). The imputed genotype matrix available for further analysis contained 31 713 SNP on the whole set of 136 accessions.

### Ecomeristem model

#### Model description

Ecomeristem is a sink-driven CGM developed for rice vegetative vigor (Luquet et al., 2006 and Luquet et al., 2007) and adapted for sorghum whole crop cycle (Larue et al., 2019). The model is implemented in C++ and is simulated following the DEVS formalism (Zeigler, 1987). The model simulates plant growth and development at organ level driven by several genotypic parameters. The model is hierarchical: it defines the organs in atomic models and their temporal interaction in coupled models.

In this study, the focus was put on sorghum biomass growth (fresh weight) during the vegetative phase. The vegetative phase plays a crucial role in determining biomass and grain yield. Mainly, during this phase sorghum plants focus on leaf area expansion which increases the plant’s ability to capture light, essential for photosynthesis, and thus contributing to biomass production and later on grain filling. Ecomeristem simulates aboveground biomass as the integration of different elementary traits: number, size and weight of the organs of each axis of the plant. The organ number is defined by an initiation rate “plastochrone, plasto_init” depending on the temperature (thermal time between the initiation of two successive phytomers). The “phyllochrone, phyllo_init” defines the time from the first growth phase of a leaf until it appears beyond the sheath of the previous leaf (thermal time between the appearances of two successive leaves). Finally, the “ligulochrone, ligulo_init” defines the time from its appearance until its ligulation (thermal time between the ligulation of two successive leaves). The size of the leaves is defined by the parameter “Meristem Growth Rate, MGR_init” which makes it possible to calculate the pre-dimensioning of the leaves under the influence of radiation (through a state variable defining the balance between supply and demand in Carbon (C), “Index of internal Competition, *IC*”). The growth of an organ is therefore defined by the final size to be reached divided by the thermal time needed for each phase (see above). Organ and whole plant growth can be slowed down if the daily supply of carbohydrates (defined by the parameter “Epsib” which converts the radiation into C) is no longer sufficient to meet the needs on the scale of the plant. The thickness of the leaves is defined by the “SLAp” parameter, which decreases the specific leaf area between successive leaves. The weight of the leaves is then defined by the leaf area divided by the specific leaf area. The “Leaf_length_to_IN_length” parameter defines the internodes’ pre-sizing compared to the corresponding leaf’s pre-sizing. The growth of the internodes then occurs as for the leaves: a final size to be reached in a defined (thermal) time, defined as being equal to 3 times the “ligulochrone”, and can be slowed down if the supply of C is not sufficient to meet the demands. Each internode’s volume and density then define the weight of the internodes. Finally, these different processes take place daily on each axis (i.e. main stem + tillers) of the plant. Tillers appear at each “plastochrone + phyllochrone” if the state of the plant (supply/demand balance in C) is favorable during the “phyllochrone” phase, that is to say if the *IC* is greater than the parameter threshold “ICt.” All of these processes therefore make it possible to integrate biomass growth during the vegetative phase at the scale of the whole plant. **Table 2** summarizes these parameters and the selected ranges of values for parameter estimation. A genotype is thus characterized by its set of genotypic parameters.

**Table 2:** Key genotypic parameters and the value ranges used for parameter estimation.

Parameter	Description (Unit)	Interval
**Epsib**	Light conversion coefficient (g.MJ^-1^)	[3.0, 8.0]
**Plasto_init/phyllo_init/ligulo_init**	Initial values of plastochron, phyllochron and ligulochron (°Cd)	[25.0, 45.0]
**Ict**	Threshold parameter tested on IC (C supply/demand ratio) enabling tillering	[0.5, 2.5]
**MGR_init**	Initial value of the additive parameter Meristem Growth Rate pre-defining the potential size of successive leaves	[6.0, 14.0]
**Leaf_length_to_IN_length**	Ratio between leaf and internode length	[0.1, 0.2]
**SLAp**	Specific leaf area decrease rate between successive leaves	[0.0, 0.1]

#### Parameter estimation of the CGM

Parameter estimation for each genotype, was performed using the Differential Evolution (DE) metaheuristic implemented in the DEoptim R package (Ardia et al., 2020). Eight key genotypic parameters were estimated (see **Table 2**) for each genotype using the data gathered on the PhenoArch platform by reducing the errors between observed and simulated values for all measured traits (see **Table 1**). The error was computed as the mean of Normalized Root Mean Square Error (NRMSE, eq. 2) over the four replicates.


(2) $$NRMSE=\sqrt{\frac{\sum_{i=1}^{n}{\left(\frac{y_{i}-{\hat{y}}_{i}}{y_{i}}\right)}^{2}}{n}}$$

Where ${\hat{y}}_{i}$ are the predicted values, $y_{i}$ the observed values and n the number of observations.

The parameter set resulting in the lowest error after 10 000 iterations of the DEoptim algorithm was then selected for each genotype, these parameter values are hereafter considered as “observed” parameter values. To facilitate the interpretation of the results, the error per observed trait is represented by a Normalized Mean Absolute Error (NMAE) in **Supplementary Table S1**.

### Genomic prediction

Genomic prediction was performed either by using the Least Absolute Shrinkage and Selection Operator (LASSO) implemented in the glmnet R package (Friedman et al., 2010), Bayes C implemented in the BGLR R package (Perez and de los Campos, 2014) or by a multilayer Convolutional Neural Networks (CNN) constructed with Tensorflow 2.0 (Singh and Manure, 2020) in Python (Van Rossum and Drake, 2009). For each method, validation was performed using a k-fold cross-validation method (with k = 5). The composition of each fold was equal across methods.

Two scenarios were investigated in this study. In the first instance, the direct prediction of aboveground fresh weight was done using the abovementioned three regression methods. The 31 713 SNP were used as the explanatory variables and the observed Biomaerofw as the explained variable. In the second scenario, the CGM Ecomeristem was used. First its input parameters were predicted using the same three regression methods, i.e. the SNP were used as the explanatory variables and the eight genotypic parameters as the explained variables. Then the predicted parameters were used to simulate growth and development of each genotype. The Ecomeristem output variable “Biomaerofw” was used as prediction for the aboveground fresh weight and compared to the observed Biomaerofw. The two scenarios and the three regression methods were compared based on the Normalized Mean Absolute Error (NMAE, Equation 3) between predicted and observed phenotypic trait, as well as the predictive ability (PA, Equation 4).


(3) $$NMAE\;\left(\%\right)=\frac{1}{n}\sum_{i=1}^{n}abs\left(\frac{y_{i-}{\hat{y}}_{i}}{y_{i}}\right)$$

(4) $$PA=\frac{Cov\left(y,\hat{y}\right)}{\sigma_{y}\sigma_{\hat{y}}}$$

Where ${\hat{y}}_{i}$ are the predicted values of either the integrative phenotypic trait or the CGM parameters, $y_{i}$ the observed values of either the trait (phenotyped on the Phenoarch platform) or the CGM parameters (estimated trough the DE optimization algorithm, see *Parameter estimation of the CGM*), σ the standard deviation and Cov the covariance.

The genetic markers used as input data were encoded as {-1, 0, 1} corresponding respectively to homozygous for the reference allele, heterozygous and the homozygous for the alternative allele.

#### LASSO

The first penalized regression method tested in this study is LASSO. This method performs variable selection and regularization and was first proposed by Tibshirani (1996). In this method, the coefficient for all SNP (β) are obtained by minimizing the sum of squares of the residuals (Equation 5), and are in addition under constraint as follows: $\sum_{j=1}^{p}\left|\beta_{j}\right|\leq t$ where *t* is an arbitrary specified parameter controlling the regularization of the estimated coefficients. This regularization is an *l*_1_-norm penalization allowing some coefficients to exactly equal zero.


(5) $$\underset{\beta_{0},\beta }{\min}\frac{1}{2}\sum_{i=0}^{n}{\left(y_{i}-\beta_{0}-\sum_{j=1}^{p}\beta_{j}x_{i,j}\right)}^{2}$$

LASSO works well in problems of high dimensionality where the number of predictors is higher than the number of individuals (p > n) but only if a few of these predictors explain the observations. Indeed, LASSO selects at most n variables before it saturates (Zou and Hastie, 2005).

#### Bayes C

The second regression method tested is Bayes C (Habier et al., 2011). This method is derived from the Bayes A and Bayes B methods (proposed by Meuwissen et al., 2001) to address some of their drawbacks. Bayes A and B have shown to better address linkage disequilibrium (LD) between SNP and QTL than with least squares of the LASSO method resulting in higher prediction accuracies (Hayes et al., 2009). However, Gianola et al. (2009) have shown certain limitations to Bayes A and B concerning the prior of marker effects.

Bayes A and B are based on the general statistical model (Equation 6). The SNP effect is zero with probability π and is normally distributed with mean zero and a locus specific variance, with probability 1-π: *N*(0, σ²_j_). In Bayes A, all SNP have non-zero effect (i.e. π = 0), while in Bayes B π > 0. Having a locus-specific variance means that the shrinkage of SNP effects heavily relies on the scale parameter. Additionally, it becomes evident that such variance introduces challenges in Bayesian learning due to the posterior having only one more degree of freedom than the prior, thus limiting the predictability of posteriors significantly deviating from the prior (Gianola et al., 2009). Bayes C has been developed to overcome these limitations by considering a common variance to all SNP (see Habier et al., 2011).


(6) $$y=µ+u+\sum_{k=1}^{K}z_{k}a_{k}+e$$

Where µ is the overall mean of the phenotypic trait, u the polygenic effects of all individuals, K is the number of SNP, $z_{k}$ the genotype at SNP k, $a_{k}$ the additive effect of that SNP and e the residual effects.

#### Convolutional neural network

**Figure 1:**
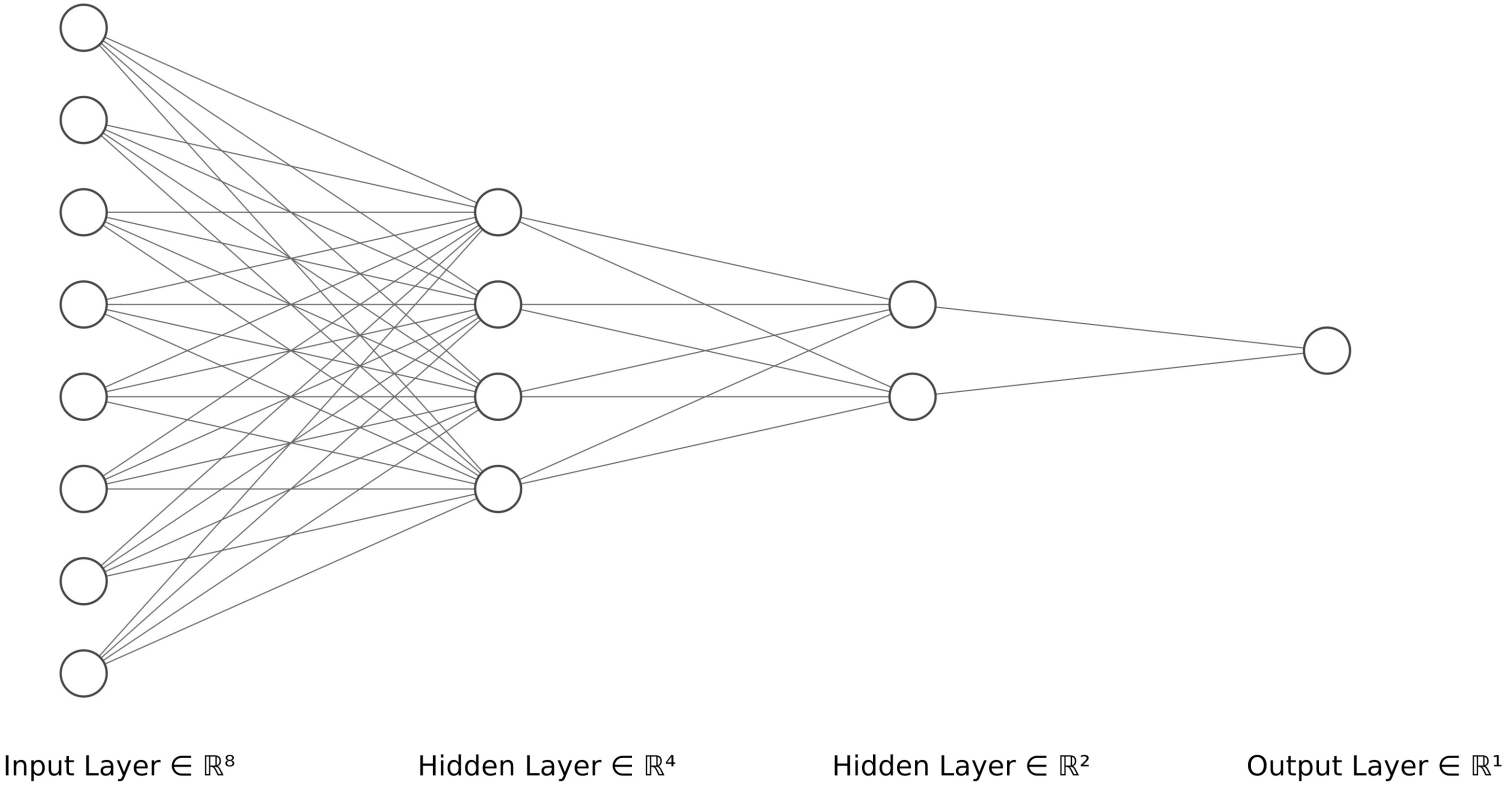
Schematic representation of a neural network with an input layer of size 8 and two hidden layers containing 4 and 2 neurons respectively. Drawn with LeNail (2019) tool.

The last method tested is a Convolutional Neural Network (CNN, LeCun et al., 2015), a type of neural network (NN). Neural networks are machine-learning methods used for regression and classification in a non-linear way. These networks are made up of a set of “neurons”, i.e. computational units, which, like neurons in the brain, can receive a signal and transmit it (through a non-linear function) to other connected neurons. These neurons are arranged in layers and the signal passes through these successive layers. Each neuron therefore performs a non-linear (and potentially local) regression of part or all the connected neurons of the previous layer (**Figure 1**).

**Figure 2:**
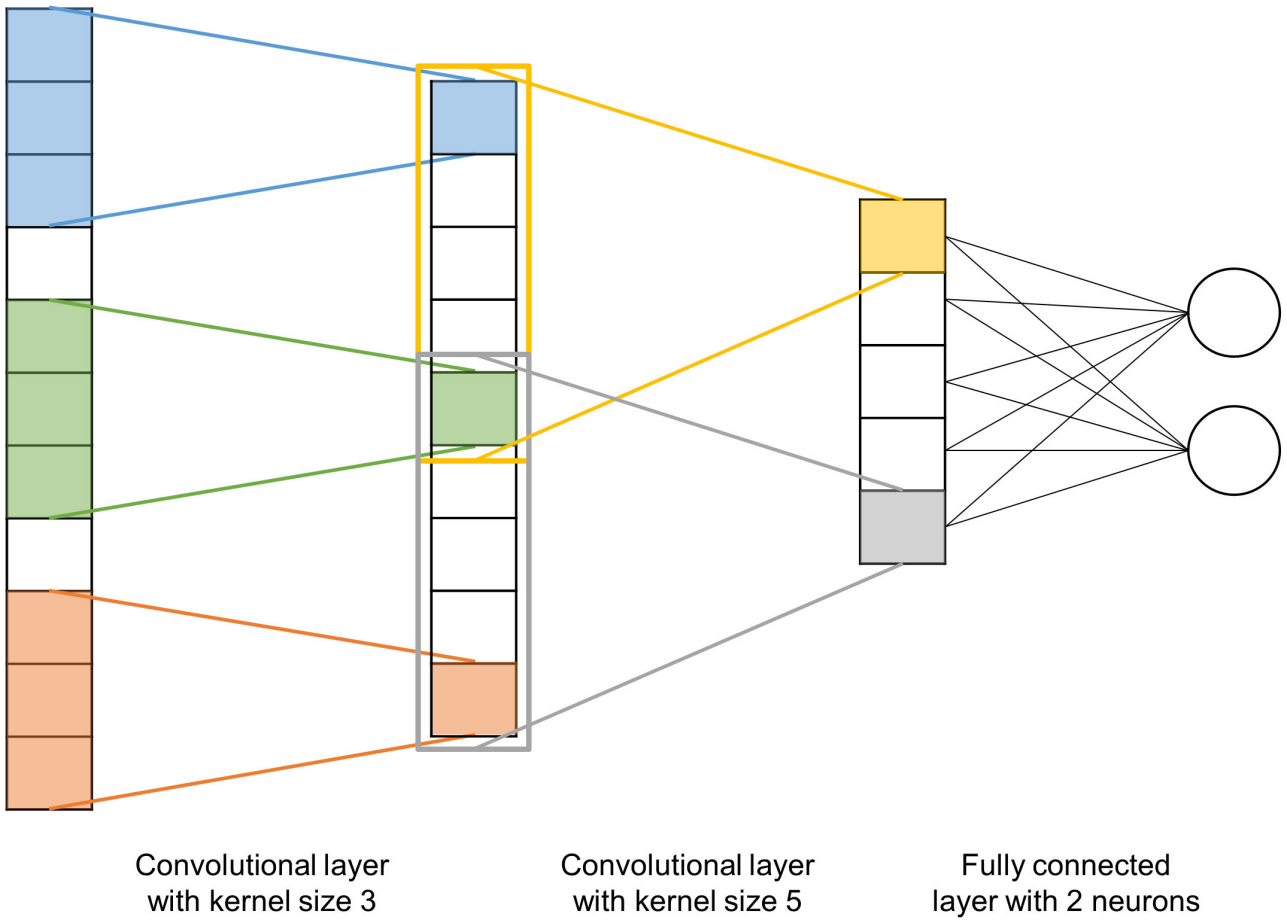
Schematic representation of a 1-dimensional convolutional neural network with two convolution layers (kernel sizes 3 and 5 respectively) and a fully connected 2-neuron layer. The kernel of the first layer (initially in blue) will slide over the input data with a step size defined by the stride (here equal to 1) and perform a convolution of the n adjacent inputs (where n is the size of the kernel). The kernel of the second layer (initially in orange) will do the same.

CNN (**Figure 2**) are a type of NN inspired by the visual cortex of the human brain where neurons respond to stimuli in specific regions of the field of view, with these regions overlapping. This principle is used in CNN through two layers: a first layer of filters that extracts high-level features (in our case, characteristics of the genetic architecture between nearby markers), this is the convolution layer that performs local regression between these markers, i.e. SNP markers effect are estimated. The second layer is the reduction of the dimension of this convolution through pooling, generally a maxpooling which returns the maximum value of the portion of markers covered by the convolution, in our case similarly to other methods considering SNP with zero effect. CNN are a continuation of these two layers of convolutions (here between markers, or non-linear regression of groups of markers) and pooling. Following these layers, CNN typically have a layer of fully connected neurons in order to learn non-linear combinations of the high-level features detected by the convolution layers. CNN have completely changed the field of image analysis because they are particularly well suited to take into account the spatial nature of data, typically the relationship between pixels in an image or similarly genetic markers along a sequence.

The CNN architecture developed in this study was designed by hyperparameter optimization (Feurer and Hutter, 2019). Hyperparameter tuning consists in trying multiple combinations of network parameters and architectures until a suitable architecture adapted to the studied data is identified. Typical hyperparameters include number of layers, size of filters, activation functions, etc. The hyperparameterized CNN was composed of five 1D-convolution layers with kernel sizes of 11, 11, 9, 13 and 9; and strides of 1, 3, 5, 5 and 5. Followed by a dense fully connected layer composed of 32 neurons, and the output layer of size 8 (number of CGM parameters to be estimated) or one (for the direct prediction of the phenotypic trait). The activation function (Nwankpa et al., 2018) between each layer was a succession Rectified Linear activation function (ReLU, Equation 7) or a softmax (Euation 8) except for the output layer where a sigmoid (Equation 9) was used. The models were trained using the Adam optimizer (Kingma and Ba, 2014) and evaluated using the NRMSE (see Equation 2) loss function.


(7) f(x)=max(0,x)

(8) $$\sigma {\left(z\right)}_{j}=\;\frac{e^{z_{j}}}{\sum_{k=1}^{K}e^{z_{k}}}$$

(9) $$f\left(x\right)=\frac{1}{1+e^{-x}}$$

### Heritability of phenotypic traits and CGM parameters

Narrow-sense heritability for all phenotypic traits, and CGM parameters, were computed using the genotypic and phenotypic data described above. In a first step, the Genomic Relationship Matrix (GRM) using the method proposed by VanRaden (2008) and implemented in the snpReady R package (Granato et al., 2018) was generated. Then, marker-based heritability was estimated using the heritability R package (Kruijer et al., 2015). The results are presented in the **Supplementary Table S2**.

## Results

### Genomic prediction of a complex integrative trait: sorghum aboveground fresh weight

**Figure 3:**
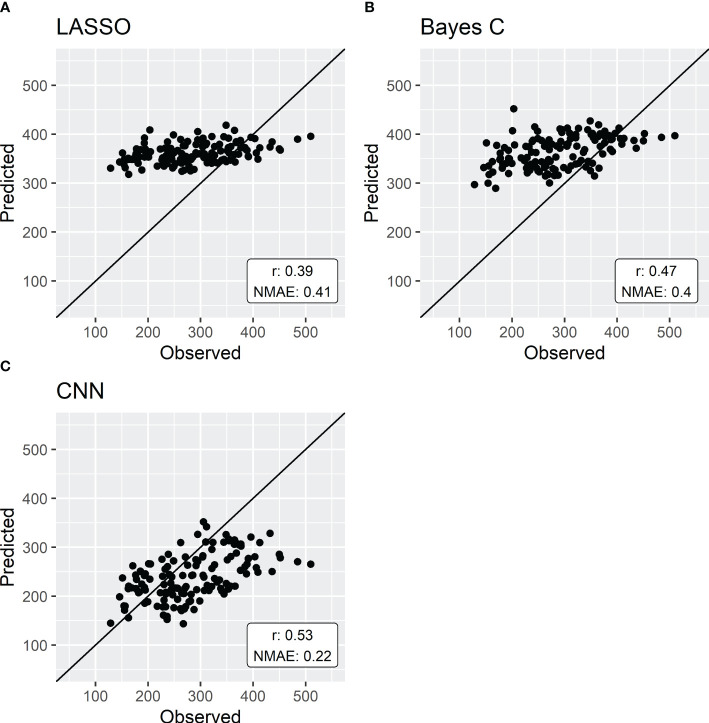
Genomic prediction of aboveground fresh weight by either LASSO **(A)**, Bayes C **(B)** or CNN **(C)**. Models are trained with a 5-fold cross-validation using data from 136 sorghum genotypes. Results are expressed by normalized mean absolute error (NMAE) and the predictive ability (r).

The first scenario compared the three regression methods on the direct prediction of sorghum aboveground fresh weight (**Figure 3**). LASSO yielded the least accurate results with a NMAE of 0.41 and a PA of 0.39. Bayes C had slightly better results with a NMAE of 0.4 and a PA of 0.47. Finally, genomic prediction using a CNN showed the best results with a NMAE of 0.22 and a PA of 0.53.

### Predicting integrative traits using genomic predicted CGM parameters

The genomic prediction of CGM parameters yielded variable results across predicted parameters but not across prediction methods (**Table 3**). NMAE varied from 0.87 for the least accurately predicted parameter, SLAp, to 0.05 for the most accurately predicted parameter, Ligulo_init. The average NMAE for each method was of 0.26 (SD = 0.27) for LASSO, 0.24 (SD = 0.24) for Bayes C and 0.24 (SD = 0.24) for CNN.

**Table 3:** Normalized Mean Absolute Error (NMAE) on the prediction of Ecomeristem parameters using LASSO, Bayes C or CNN.

RMSE	Epsib	Ict	MGR_init	Plasto_init	Phyllo_init	Ligulo_init	LL_to_IL	SLAp
LASSO	0.19	0.28	0.09	0.15	0.07	0.05	0.38	0.87
Bayes C	0.18	0.27	0.09	0.15	0.07	0.05	0.33	0.78
CNN	0.17	0.26	0.09	0.14	0.07	0.05	0.33	0.79

**Figure 4:**
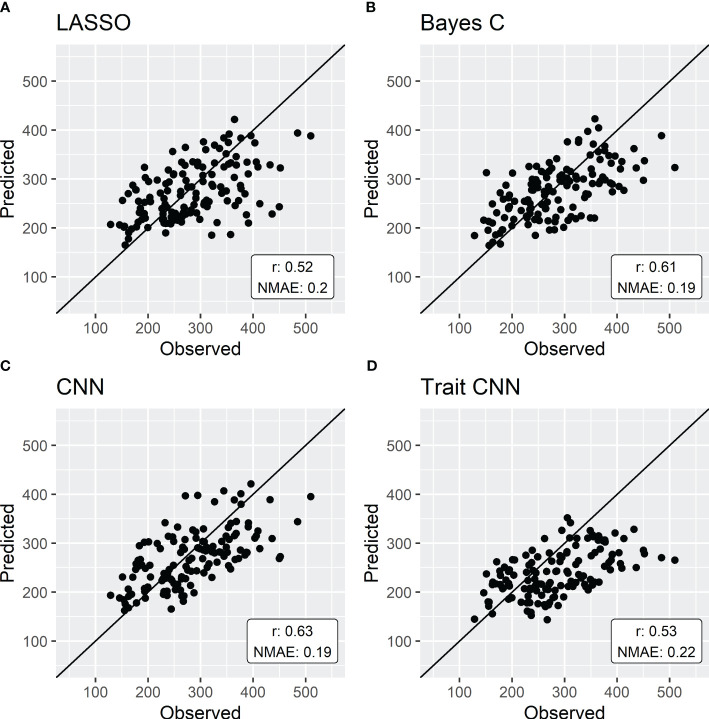
**|** 5-fold cross validation of simulations of Biomaerofw by Ecomeristem using parameters predicted by either LASSO **(A)**, Bayes C **(B)** or CNN **(C)** compared to the direct genomic prediction of the integrative trait aboveground fresh weight **(D)**. Each point represents the mean predicted value of the genotypes forming the validation set in each fold.

The prediction of Biomaerofw using Ecomeristem (**Figure 4**) was slightly more precise when using parameters predicted with a CNN (NMAE 0.19, PA 0.63). LASSO and Bayes C still yielded parameters that resulted in good predictions (NMAE 0.20 and 0.19 respectively and PA 0.52 and 0.61).

Biomass components (secondary traits) were also simulated using Ecomeristem, based on the prediction of genotypic parameters through a convolutional neural network and are presented in **Supplementary Figure 1**. Mainstem biomass (Mainstemfw, NMAE = 0.18, r = 0.72) as well as plant height (Pht, NMAE = 0.18, r = 0.6) were accurately predicted. The predictive ability for both individual leaf size (Arealfel, NMAE = 0.13, r = 0.53) and leaf biomass (Bladefw, NMAE = 0.22, r = 0.47) were lower.

## Discussion

In the present study, prediction of an integrative trait, sorghum aboveground fresh weight (Biomaerofw), using different methods was analyzed. The crop growth model Ecomeristem was then used to better consider the direct environmental effects and their interaction with genotypes on biomass growth. Eight genotypic parameters were predicted using the same three regression methods. Finally, the CGM predictions were compared to the direct genomic prediction of Biomaerofw.

### Convolutional neural networks enhanced prediction accuracy of integrative traits in direct genomic prediction

Integrative traits are under the influence of (i) many genes and genetic interactions, especially epistasis (Zuk et al., 2012), and (ii) Genotype x Environment interactions (Rincent et al., 2017). These interactions are, by nature, non-linear. Therefore, to assess the capacity of linear regression models in producing accurate predictions of these traits, LASSO and Bayes C were compared to a non-linear regression method, specifically a convolutional neural network (CNN). The results revealed that LASSO exhibited the least accurate predictions for the studied integrative trait. While Bayes C demonstrated higher prediction accuracy, it still faced challenges in predicting aboveground fresh weight compared to convolutional neural networks (almost twice as high NMAE, **Figure 3**). These results are in line with previous studies comparing LASSO and Bayesian methods (e.g. Howard et al., 2014).

The use of non-linear regression methods, capable of efficiently considering interactions between polymorphism sites, could potentially address the challenge of epistasis in predicting integrative traits. Results showed that CNN, as a non-linear regression method, surpassed both LASSO and Bayes C in predicting total biomass fresh weight. The complexity of sorghum biomass growth, influenced by numerous interdependent biological processes and characterized as polygenic (Habyarimana et al., 2020), underscores the relevance of considering epistatic interactions. Previous studies have shown the influence of epistasis on complex trait variation, such as growth rate or crop yield (Kroymann and Mitchell-Olds, 2005; Melchinger et al., 2007). Given that integrative traits are influenced by myriad of local and distal SNP-SNP interactions (Qian et al., 2017), convolutional neural networks, by design, emerge as a suitable tool for accommodating these epistatic interactions in the prediction of these traits. Recent studies proposed the application of neural networks (NN), and in particular CNNs in genomic prediction of complex human and animal traits (e.g. Pook et al., 2020). These studies show that NNs can exhibit either slightly better, similar or inferior prediction accuracies compared to classical linear regression. Notably, the outcome is highly dependent on the set of SNPs, the genetic architecture of the trait (e.g. Bellot et al., 2018), and the architecture of the NN itself (Zhu et al., 2021).

The use of NNs in genomic prediction is obviously case-dependent, and it is apparent that more research is needed to adapt these non-linear methods to the Genomic Selection process. Specifically, this involves understanding how genetic data is processed by the NN, and assessing their impact on prediction outcomes (e.g. Verplaetse et al., 2023). In contrast to classical linear regression, a universal neural network architecture that suits all cases does not exist. Apart from estimating regression parameters, the entire structure (including the number, type, and size of layers, activation functions, etc.) must be adapted to the specific prediction problem at hand.

### Successful integration of crop growth models in genomic prediction of integrative traits

The Ecomeristem crop growth model was used to provide a more comprehensive consideration of causal non-linear environmental effects, as well as interactions between biomass components on biomass growth, potentially leading to enhanced prediction accuracies. Biomass is a highly polygenic trait, Habyarimana et al. (2020) detected significant marker-trait associations across eight of the then sorghum chromosomes. It is also under the influence of epistatic interactions: Brown et al. (2008) highlighted epistatic interactions between two major dwarfing QTL, Ishimori et al. (2020) showed the important role of epistasis for total biomass as well as stem length. Moreover, biomass components show differences in the dynamics of the effects of different genes depending on environmental conditions as well as developmental stage (Mu et al., 2022).

In this context, we proceeded to predict genotypic parameters that govern the equations formalizing biological processes where GxE, as well as interactions between resulting intermediate traits take place. Not all CGM parameters where predicted with the same accuracy. The least accurate predicted parameter was associated with the control of specific leaf area decrease between successive leaves (SLAp), with an average NMAE of 0.81. This can be attributed to the limited variability observed on this parameter across genotypes, with 40% of them having identical values, although SLAp exhibited high heritability (0.616). The challenge in accurately predicting SLAp emphasizes the influence of parameter characteristics and parameter estimation quality on the efficiency of genomic predictions of crop model parameters. Variations in prediction accuracy across other parameters can be explained by their heritability (see **Supplementary Table S2**). Parameters such as Plasto_init, Phyllo_init and Ligulo_init, characterized by a lower heritability, resulted in less accurate predictions. Conversely, parameters with higher heritability, including MGR_init, LL_to_INL, and ICt, exhibited predictions that are more accurate.

While errors are not directly comparable between parameters, intermediate traits, and the integrative trait, the simulation of Biomaerofw with Ecomeristem showed higher accuracies than the direct prediction of the trait, as illustrated in **Figures 4C** and **D**. This observation aligns with recent studies (e.g. Heslot et al., 2014; Tolhurst et al., 2022; Filho et al., 2023) which highlighted the enhanced prediction accuracies achieved through the incorporation of environmental effects. Interestingly some studies, like the one of Widener et al. (2021), suggest that the inclusion of environmental covariates may have limited impact, if any, on improving predictions in extreme environments. Moreover, they suggest that the composition of the calibration set of environments may hold greater significance as they found that only a subset of the available environments was needed to accurately predict GEBV. In addition, Rogers and Holland (2022) showed that the environmental similarity between training and test sets had a great impact on prediction accuracies. Phenomic selection (PS, Rincent et al., 2018) is one way of considering the impact of GxE. It is suggested that environmental variation is captured by the spectra and could thus enhance prediction accuracies compared to genomic prediction (e.g. Lane et al., 2020; Robert et al., 2022). On the other hand, the integration of crop models, as proposed in our study, introduces promising avenue to include causal environmental effects into GP. By providing causal relationships between environmental variation and individual traits, crop models have the potential to offer insights of the environmental effect on phenotypic plasticity. For example, the Ecomeristem model has already demonstrated its ability to predict plant behavior in new environments, i.e. not used during the estimation of genotypic parameters, or for alternate crop management (Larue et al., 2019). In addition, CGM simulate integrative traits as a result of interactions between intermediate traits and as responses to plant state. Therefore, the integrated CGM-GP approach could also answer the varying effects of genes depending on plant developmental stage. However, further research is imperative to thoroughly assess and quantify the impact of the integration of crop models with genomic prediction on prediction accuracy of the genetic values of candidates to selection, particularly in the context of multi-environmental trials. Our dataset was composed of a single environment. While GxE was still introduced by considering individual plant micro-environment inside the greenhouse (see Phenotypic data section), it is still necessary to validate this approach in a multi-environment setting. Several studies have explored this question (e.g. Reymond et al., 2003; Technow et al., 2015; Jighly et al., 2023). Nevertheless, it is important to note that their methodologies differ from the approach proposed in our study, particularly in terms of how crop model parameters are predicted through the use of genomic prediction: in their approach, genotypic parameters are sampled in each iteration of a Bayesian model and used for predicting observed traits through the CGM that is then used to update the marker effects. They are thus estimating parameters at the same time as the markers effects. In our approach, the parameter values are considered to be known. More specifically, the parameters are estimated in a preliminary step by model inversion using an optimization algorithm (here, a metaheuristic called Differential Evolution). These “observed” parameter values are then used to train a prediction model using marker information with no feedback of the CGM performance, relying solely on the difference between predicted and “observed” parameter values. Once trained, the prediction model is then used to predict the value of these genotypic parameters for the validation set and are then fed to the CGM to predict the phenotypic traits. Furthermore, these studies often focus on either a limited number of markers or crop model parameters; or they consider a reduced set of physiological processes within the crop model. The differences in methods and areas of interest highlights the need for a comprehensive study into the potential benefits of employing crop models for predicting integrative traits.

As mentioned previously, the prediction accuracy of the CGM-GP approach is highly dependent on the calibration set, the quality of parameter estimation and also the relevance of the crop model (Rincent et al., 2017). Indeed, the main limiting factor of the CGM-GP approach is how well the crop model is able to simulate the integrative trait of interest. In our study, the “optimal” parameter set yielded an average NMAE of 0.065 on Biomaerofw (**Supplementary Table S1**). A perfect prediction of the CGM parameters could not yield a more precise result than the “optimal” parameter set. In any case, our study shows that the CGM-GP approach improves the predictive accuracy of an integrative trait compared to its direct prediction.

### Comparative analysis: linear and non-linear regression methods yield similar and robust results when predicting crop growth model parameters

In this study, we showed that using non-linear regression methods (convolutional neural networks) in the genomic prediction process yielded higher prediction accuracies than classical linear methods, such as LASSO or Bayes C. However, CNN superiority is less significant when the prediction target is CGM parameters rather than the integrative trait (**Figure 3**). **Table 3** shows that, across all CGM parameters, the NMAE is similar for all regression methods. The same trend in parameter prediction accuracy as highlighted before is true for the three regression methods. As mentioned previously, it is suggested that the crop models decompose integrative traits into potentially more heritable intermediate traits (organ size, number, etc.) that are simulated through response curves to the environment, defined by genotypic parameters and can thus be used to predict the genetic variability of these integrative traits (Reymond et al., 2003; Parent and Tardieu, 2014). Indeed, the heritability of the integrative trait Biomaerofw was of 0.714 while the elementary traits composing aboveground biomass systematically harbored higher heritability with the exception of the number of tillers (see **Supplementary Table S2**). Most of these traits were indeed simulated with higher accuracy than the direct prediction of aboveground biomass (see **Supplementary Figure S1**).

It is interesting to note that the prediction of the trait through a CGM is also more accurate for the LASSO and Bayes C methods compared to the direct prediction of the trait. These results may show that genotypic parameters are under less complex genetic control than the integrative traits of interest. It could also indicate the robustness of the crop model for parameter sets that deviate slightly from the observed values. Lastly, considering causal effects of the environment on intermediate traits, as formalized in crop models, could enhance prediction accuracies of the integrative trait.

## Conclusion

The present study suggested that the use of convolutional neural networks (CNN) to predict complex integrative phenotypic traits enhanced prediction accuracies in classical Genomic Prediction approaches by considering non-linear genetic interactions. It also reaffirmed the benefits of using crop growth models (CGM) to better account for environmental effects on these traits as well as facilitating marker-based prediction by breaking down integrative traits into simpler traits. Epistatic genetic architecture and non-linear relationships between traits and between CGM parameters were suggested as the explanation for the more robust results of CNN. The CGM-GP approach using CNN showed promising results in a multi-trait (MT) context and could also enhance predictions in a multi-environment (ME) and MTME context.

## Data availability statement

The original contributions presented in the study are publicly available. This data can be found here: https://github.com/GBeurier/GenomicPrediction_Frontier.

## Author contributions

FL: Conceptualization, Data curation, Formal analysis, Investigation, Methodology, Software, Validation, Visualization, Writing – original draft, Writing – review & editing. LR: Conceptualization, Data curation, Formal analysis, Investigation, Methodology, Software, Supervision, Validation, Visualization, Writing – original draft, Writing – review & editing. DP: Conceptualization, Data curation, Methodology, Supervision, Validation, Writing – original draft, Writing – review & editing. J-FR: Conceptualization, Data curation, Methodology, Supervision, Writing – original draft, Writing – review & editing. DL: Conceptualization, Data curation, Formal analysis, Funding acquisition, Investigation, Methodology, Project administration, Supervision, Validation, Visualization, Writing – original draft, Writing – review & editing. GB: Conceptualization, Data curation, Formal analysis, Investigation, Methodology, Software, Supervision, Validation, Visualization, Writing – original draft, Writing – review & editing.

## Supplementary material

Supplementary Figure 1 5-fold cross validation of simulations of mainstem fresh weight **(A)**, plant height **(B)**, area of last fully elongated leaf **(C)** and mainstem leaf blade fresh weight **(D)** by Ecomeristem using genomic predicted parameters obtained with the use of a convolutional neural network.

Supplementary Table 1 Results of parameter estimation expressed as the mean of normalized mean absolute error over all genotypes for each observed phenotypic trait.

Supplementary Table 2 Narrow sense heritability for estimated parameters and their corresponding trait.

Supplementary Table 3 Normalized Mean Absolute Error (NMAE) and Predictive Ability (PA) for the three genomic prediction model used either in the direct prediction of sorghum biomass (direct-GP) or the prediction through an integrated crop growth model – genomic prediction (CGM-GP) approach.
