# Supplementary figures and images for: Linking genetic markers and crop model parameters using neural networks to enhance genomic prediction of integrative traits
Source: Front Plant Sci. 2024 Jul 30;15:1393965. doi: 10.3389/fpls.2024.1393965 (PMC11319263; doi:10.3389/fpls.2024.1393965)

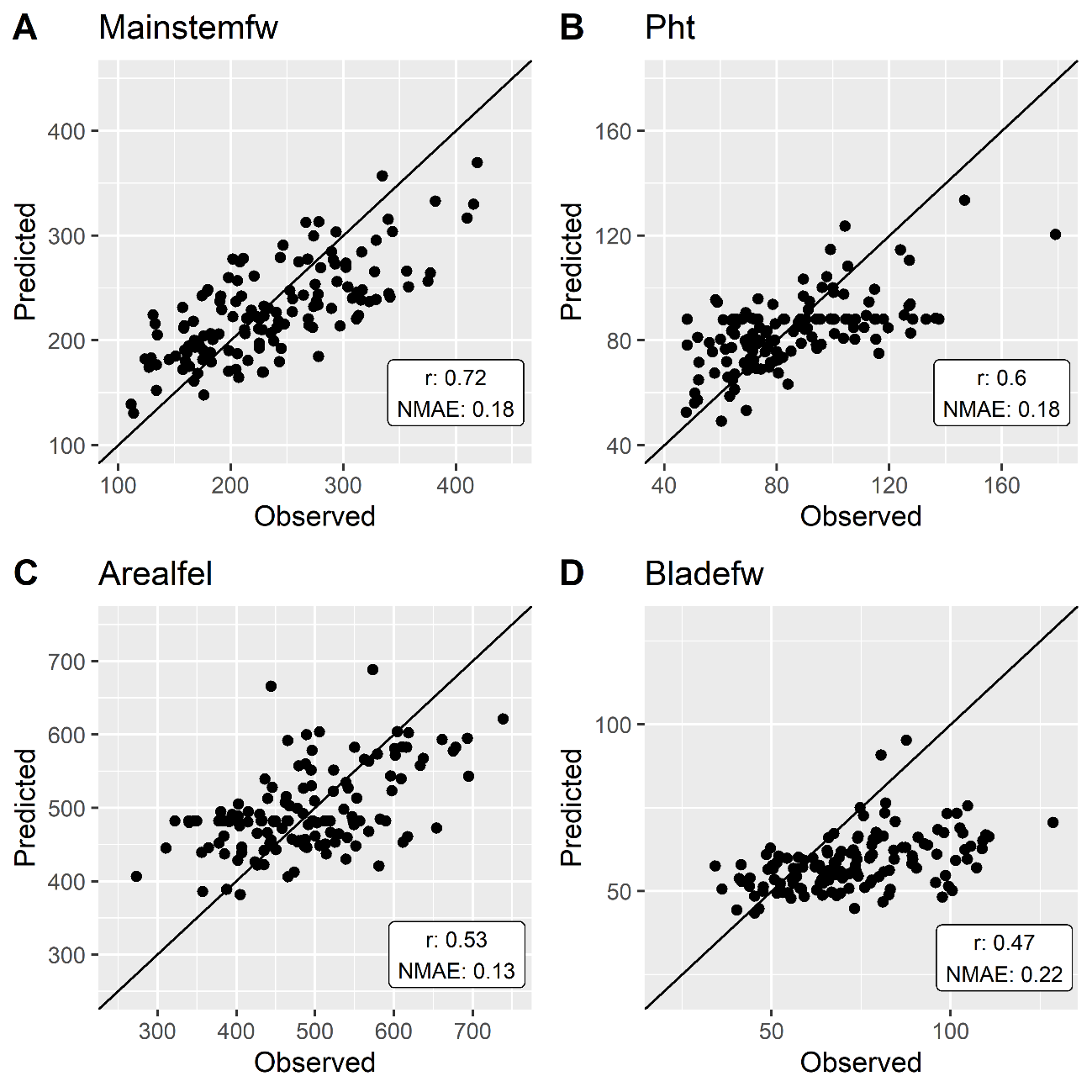

Supplement: Supplementary Figure 1 — 5-fold cross validation of simulations of mainstem fresh weight (A), plant height (B), area of last fully elongated leaf (C) and mainstem leaf blade fresh weight (D) by Ecomeristem using genomic predicted parameters obtained with the use of a convolutional neural network. [file Image_1.tif]
