# Supplementary material for: Linking genetic markers and crop model parameters using neural networks to enhance genomic prediction of integrative traits
Source: Front Plant Sci. 2024 Jul 30;15:1393965. doi: 10.3389/fpls.2024.1393965 (PMC11319263; doi:10.3389/fpls.2024.1393965)
Supplement: Supplementary Table 1 — Results of parameter estimation expressed as the mean of normalized mean absolute error over all genotypes for each observed phenotypic trait. [file Table_1.docx]

**Table S.1: Results of parameter estimation expressed as the mean of normalized mean absolute error over all genotypes for each observed phenotypic trait**

|  | NMAE |
| --- | --- |
| App | 0.056 |
| Biomaerofw | 0.065 |
| Mainstem | 0.085 |
| Lig | 0.120 |
| Pht | 0.120 |
| Arealfel | 0.053 |
| Tillernb | 0.136 |
