# Supplementary material for: Linking genetic markers and crop model parameters using neural networks to enhance genomic prediction of integrative traits
Source: Front Plant Sci. 2024 Jul 30;15:1393965. doi: 10.3389/fpls.2024.1393965 (PMC11319263; doi:10.3389/fpls.2024.1393965)
Supplement: Supplementary Table 2 — Narrow sense heritability for estimated parameters and their corresponding trait. [file Table_2.docx]

**Table S.2: Narrow sense heritability for estimated parameters and their corresponding trait**

| **Param / Trait** | **Parameter h²** | **Trait h²** |
| --- | --- | --- |
| Epsib / NA | 0.350 | NA |
| Ict / Tillernb | 0.951 | 0.497 |
| MGR_init / Leaflen | 0.827 | 0.840 |
| Plasto_init / NA | 0.083 | NA |
| Phyllo_init / App | 0.409 | 0.787 |
| Ligulo_init / Lig | 0.141 | 0.939 |
| Leaf_length_to_IN_length / Pht | 0.924 | 0.933 |
| SLAp / Leaflen | 0.616 | 0.840 |
| NA / Biomaerofw | NA | 0.714 |
