# Supplementary material for: Linking genetic markers and crop model parameters using neural networks to enhance genomic prediction of integrative traits
Source: Front Plant Sci. 2024 Jul 30;15:1393965. doi: 10.3389/fpls.2024.1393965 (PMC11319263; doi:10.3389/fpls.2024.1393965)
Supplement: Supplementary Table 3 — Normalized Mean Absolute Error (NMAE) and Predictive Ability (PA) for the three genomic prediction model used either in the direct prediction of sorghum biomass (direct-GP) or the prediction through an integrated crop growth model – genomic prediction (CGM-GP) approach. [file Table_3.docx]

**Table S.3: Normalized Mean Absolute Error (NMAE) and Predictive Ability (PA) for the three genomic prediction model used either in the direct prediction of sorghum biomass (direct-GP) or the prediction through an integrated crop growth model – genomic prediction (CGM-GP) approach**

|  | **Direct-GP** | | **CGM-GP** | |
| --- | --- | --- | --- | --- |
| **Model** | **NMAE** | **PA** | **NMAE** | **PA** |
| LASSO | 0.41 | 0.39 | 0.20 | 0.52 |
| Bayes C | 0.40 | 0.47 | 0.19 | 0.61 |
| CNN | 0.22 | 0.53 | 0.19 | 0.63 |
